# Concomitant inflammation/metabolism transcriptional regulatory networks in liver disease

**DOI:** 10.17179/excli2020-1056

**Published:** 2020-01-27

**Authors:** Tahany Abbas, Walaa Murad, Reham Hassan

**Affiliations:** 1 Histology Department, Faculty of Medicine, South Valley University, Qena 83523, Egypt; 2 Department of Forensic Medicine and Toxicology, Faculty of Veterinary Medicine, South Valley University, Qena 83523, Egypt

## ⁯⁯⁯

***Dear Editor, ***

Recently, Campos and colleagues published a comprehensive study about transcriptional regulatory networks in liver disease (Campos et al., 2020[1]). The authors performed a comprehensive genome-wide study including data of mouse liver tissue at eight time periods after acute CCl_4_ injury. Moreover, acute damage after lipopolysaccharide as well as tunicamycin exposure were studied and the translational relevance was examined by comparing the findings to expression changes in human liver disease (Campos et al., 2020[1]). A key observation made in all mouse and human liver diseases was the concomitant regulation of inflammatory and metabolic genes, where inflammation-associated factors are up and metabolism-associated genes downregulated. Importantly, the same upstream regulators are involved in this response so that increased inflammation and suppressed metabolism occur within one intertwined regulatory network (Campos et al., 2020[1]).

Upon severe damage, the liver is able to regenerate more than 70 % of its mass (Godoy et al., 2013[5]). However, this regenerative process represents a major challenge that requires architectural reorganization (Hoehme et al., 2010[8]; Schliess et al., 2014[13]; Vartak et al., 2016[14]) as well as the activation of massive transcriptional programs (Godoy et al., 2016[6]; Zellmer et al., 2010[15]; Ghallab et al., 2019[4]; Grinberg et al., 2014[7]; Leist et al., 2017[11]). Inflammation is known to support tissue repair by elimination of the causes of injury (Karin and Clevers, 2016[10]; Campos et al., 2014[2]) and a link between inflammatory and regenerative responses has already been described (Michalopoulos, 2013[12]; Hwang et al., 2019[9]; Fortier et al., 2019[3]). The relevance of suppressing mature liver functions, such as metabolism during inflammation, is not yet understood but a possibility is that this response helps to focus more cellular resources on regeneration. It will be interesting to learn in future if the inflammation-associated suppression of mature organ functions occurs only in liver or represents a general feature of tissue regeneration. 

## Conflicts of interest

The authors declare no conflict of interest.
